# The Effect of Size, Maturation, Global Asphyxia, Cerebral Ischemia, and Therapeutic Hypothermia on the Pharmacokinetics of High-Dose Recombinant Erythropoietin in Fetal Sheep

**DOI:** 10.3390/ijms21093042

**Published:** 2020-04-25

**Authors:** Simerdeep K. Dhillon, Guido Wassink, Christopher A. Lear, Joanne O. Davidson, Nicholas H.G. Holford, Alistair J. Gunn, Laura Bennet

**Affiliations:** 1 The Department of Physiology, The University of Auckland, Auckland 1023, New Zealand; s.dhillon@auckland.ac.nz (S.K.D.); g.wassink@auckland.ac.nz (G.W.); christopher.lear@auckland.ac.nz (C.A.L.); joanne.davidson@auckland.ac.nz (J.O.D.); aj.gunn@auckland.ac.nz (A.J.G.); 2 The Department of Pharmacology and Clinical Pharmacology, The University of Auckland, Auckland 1023, New Zealand; n.holford@auckland.ac.nz

**Keywords:** erythropoietin, pharmacokinetics, fetal sheep, allometric size scaling, maturation of elimination, asphyxia, hypoxic-ischemia, therapeutic hypothermia

## Abstract

High-dose human recombinant erythropoietin (rEPO) is a promising potential neuroprotective treatment in preterm and full-term neonates with hypoxic-ischemic encephalopathy (HIE). There are limited data on the pharmacokinetics of high-dose rEPO in neonates. We examined the effects of body weight, gestation age, global asphyxia, cerebral ischemia, hypothermia and exogenous rEPO on the pharmacokinetics of high-dose rEPO in fetal sheep. Near-term fetal sheep on gestation day 129 (0.87 gestation) (full term 147 days) received sham-ischemia (*n* = 5) or cerebral ischemia for 30 min followed by treatment with vehicle (*n* = 4), rEPO (*n* = 8) or combined treatment with rEPO and hypothermia (*n* = 8). Preterm fetal sheep on gestation day 104 (0.7 gestation) received sham-asphyxia (*n* = 1) or complete umbilical cord occlusion for 25 min followed by *i.v.* infusion of vehicle (*n* = 8) or rEPO (*n* = 27) treatment. rEPO was given as a loading bolus, followed by a prolonged continuous infusion for 66 to 71.5 h in preterm and near-term fetuses. A further group of preterm fetal sheep received repeated bolus injections of rEPO (*n* = 8). The plasma concentrations of rEPO were best described by a pharmacokinetic model that included first-order and mixed-order elimination with linear maturation of elimination with gestation age. There were no detectable effects of therapeutic hypothermia, cerebral ischemia, global asphyxia or exogenous treatment on rEPO pharmacokinetics. The increase in rEPO elimination with gestation age suggests that to maintain target exposure levels during prolonged treatment, the dose of rEPO may have to be adjusted to match the increase in size and growth. These results are important for designing and understanding future studies of neuroprotection with high-dose rEPO.

## 1. Introduction

Erythropoietin is a hematopoietic cytokine used for treatment and prevention of anemia in preterm neonates [1]. Preclinical and clinical studies have examined the potential neuroprotective and neuroreparative effect of human recombinant erythropoietin (rEPO) after hypoxic-ischemia (HI) [2,3,4,5,6,7]. Neuroprotection with rEPO treatment after HI is associated with anti-apoptotic, anti-inflammatory and neuroreparative mechanisms [3,8,9]. In P7 rats, neuroprotection with rEPO after HI (Rice–Vannucci occlusion model, 8% O_2_ for 1.5 h) showed a U-shaped dose–response curve, and repeated doses were required to achieve optimal neuroprotection [10]. rEPO treatment (5000 IU/kg, s.c.), starting immediately after HI and repeated daily for three days, was the most effective for reducing cerebral tissue loss assessed one week after HI [10]. This dose achieved an average peak plasma rEPO concentration from 6224 (s.c.) to 10,000 IU/L (i.p.) and an average area under the curve over 48 h after a single dose (AUC_0–48_) from 117,677 (s.c) to 140,000 IU/L*h (i.p) [11]. These exposure metrics were proposed as the potential targets for rEPO neuroprotection for clinical trials in neonates [12,13,14]. The optimal dose regimen and start time of treatment required for neuroprotection with rEPO remain unclear [8].

Phase I/II trials in term neonates with hypoxic-ischemic encephalopathy (HIE) reported a dose-dependent non-linear clearance of rEPO, and established that a single intravenous dose of 1000 IU/kg achieved an average rEPO plasma concentration AUC_0–48_ of 114,180 IU /L*h that was comparable to the exposure in neonatal rats [12,13,14]. Randomized control trials in term infants with HIE have examined neuroprotection with treatment with repeated boluses of high dose rEPO (1000–2500 IU/Kg) [2,4,15,16]. Similarly, studies in preterm neonates have examined prophylactic treatment with high-dose rEPO (1000–3000 IU/Kg) for improving neurodevelopmental outcomes [9,17].

Although clinical trials have already been undertaken, the pharmacokinetics of high-dose rEPO in neonates are not completely understood. There are limited data on the modulating effects of exogenous rEPO exposure, gestation age, exposure to HI, and therapeutic hypothermia treatment on rEPO pharmacokinetics. A recent study in preterm human neonates proposed that the elimination of rEPO is attributable to saturable binding of rEPO to EPO receptors (EPO-R) along with a parallel first-order elimination pathway [18]. However, the effect of maturation or rEPO exposure-associated changes in EPO-R concentration and elimination were not examined in that study. Full-term neonates with HIE receiving combined treatment with therapeutic hypothermia and rEPO (1000 IU/kg) had lower clearance of rEPO than that reported in preterm neonates given prophylactic rEPO (1000 IU/kg) [4,12,13]. Lower clearance in term neonates was primarily thought to be associated with therapeutic hypothermia [4], but the effects of exposure to global HI could not be assessed. 

A better understanding of the pharmacokinetics of high-dose rEPO is important for further clinical development and for designing preclinical studies to compare various treatment regimens of rEPO. We have recently completed studies in preterm (0.7 gestation, brain maturation equivalent to 28–32 week preterm neonates) and near-term (0.87 gestation, equivalent to term neonates) fetal sheep examining neuroprotection with rEPO treatment administered as prolonged continuous infusion independently or as a combined treatment with therapeutic hypothermia after HI [7,19]. Collectively, these studies showed that treatment with a prolonged continuous infusion of rEPO after HI reduced neuronal loss in both preterm and near-term fetal sheep [7,19]. Here, we report an analysis of pharmacokinetic models of combined first-order and mixed-order elimination, first-order and saturable EPO-R-mediated clearance and rEPO treatment-induced changes in EPO-R to describe rEPO elimination. We examined the effect of an increase in fetal size, maturation, exposure to global asphyxia, cerebral ischemia, therapeutic hypothermia and exogenous rEPO treatment on the pharmacokinetics of high-dose rEPO in well-established fetal sheep models [7,19,20,21,22]. Finally, we compared the pharmacokinetics of high-dose rEPO in preterm fetal sheep given an *i.v.* bolus dose to the reported population pharmacokinetics in preterm neonates treated with repeated boluses of high-dose rEPO [9,12]. 

## 2. Results

### 2.1. rEPO Plasma Concentration Profile

**Figure 1:**
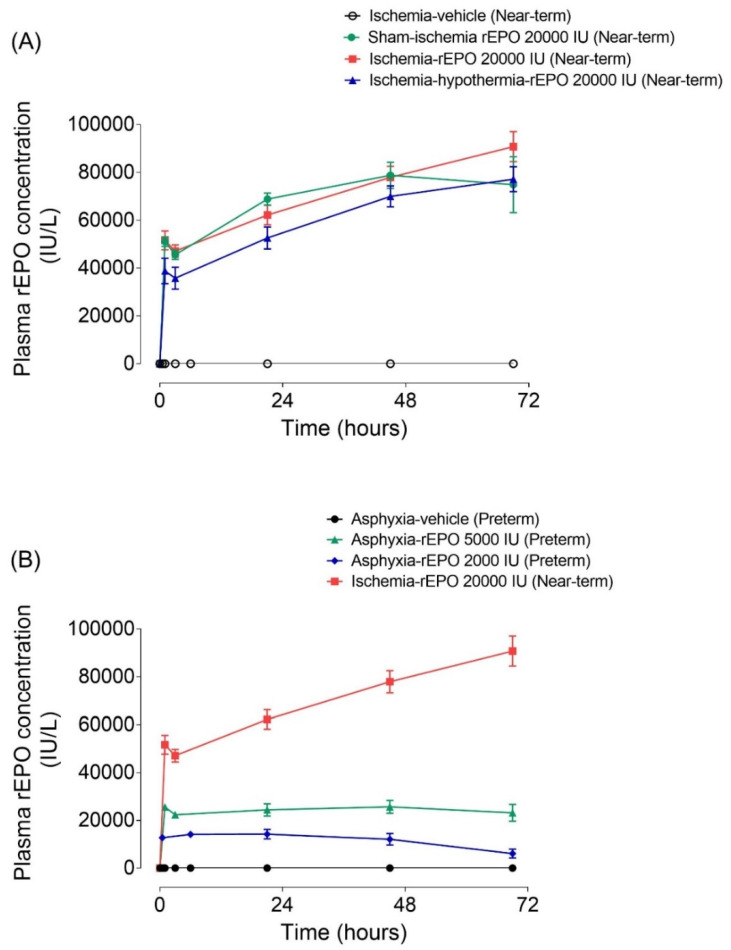
Panel **A**: Time sequence of plasma concentrations of recombinant erythropoietin (rEPO, IU/L)) in the near-term ischemia-vehicle (open-circles) (*n* = 3), sham-ischemia-rEPO 20,000 IU (closed circles, green) (*n* = 5), ischemia-rEPO 20,000 IU (rectangle, red) (*n* = 8), and ischemia-hypothermia-rEPO 20,000 IU (triangle, blue) (*n* = 8) groups at 1 h pre-EPO and 1, 3, 21, 45 and 69 h during rEPO infusion. rEPO was administered as a loading dose of 20,000 IU, based on estimated fetal weight of 4 kg, followed by a continuous infusion at 3333.3 IU/h. Panel **B**: Time sequence of plasma concentrations of rEPO (IU/L) in preterm asphyxia-vehicle (circles, black) (*n* = 8), asphyxia-rEPO 2000 IU (diamond, blue) (*n* = 8), asphyxia-rEPO 5000 IU (triangle, green) (*n* = 8) groups, and in the near-term ischemia-rEPO 20,000 IU (rectangle, red) (*n* = 8) group at 1 h pre-EPO, 30 min, 1, 3, 21, 45 and 69 h during rEPO infusion. In the preterm groups, rEPO was administered as a loading dose of 5000 IU followed by a continuous infusion at 833.3 IU/h or a loading dose of 2000 IU and infusion at 520 IU/h, based on an estimated fetal weight of 1kg. rEPO, recombinant erythropoietin.

In all vehicle-treated fetuses, rEPO plasma concentration remained below the lower limit of detection of the assay (Figure 1). rEPO plasma concentrations in the near-term sham-ischemia rEPO infusion, ischemia-rEPO infusion, and ischemia-hypothermia rEPO infusion groups are shown in Figure 1. Collectively, these results demonstrate that exposure to cerebral ischemia or therapeutic hypothermia in near-term groups did not detectably alter rEPO plasma concentrations. The pilot experiments in preterm fetal sheep showed comparable rEPO plasma concentrations in sham-asphyxia rEPO infusion (*n* = 1) and asphyxia-rEPO infusion-treated animals (*n* = 3) (Supplementary Materials Figure S1). Average rEPO plasma concentrations in the near-term rEPO and preterm rEPO groups are shown in Supplementary Table S1.

### 2.2. rEPO Bolus Treatment in Preterm Fetal Sheep

The average (±SEM) plasma concentration at 30 min after a 5000 IU bolus dose of rEPO was 27,680 ± 2355 IU/L. The single dose AUC of rEPO plasma concentration over 48 h after the first rEPO bolus dose was 110,998 ± 7027 IU/L*h. The AUC was calculated by integration of the predicted rEPO plasma concentration. (Supplementary Materials Figure S2).

### 2.3. rEPO Pharmacokinetics

There were 65 baseline measurements less than the limit of detection and these were excluded from the analysis. A total of 446 measured concentrations above the limit of detection were used in a combined analysis of all groups of fetal sheep. 

**Figure 2:**
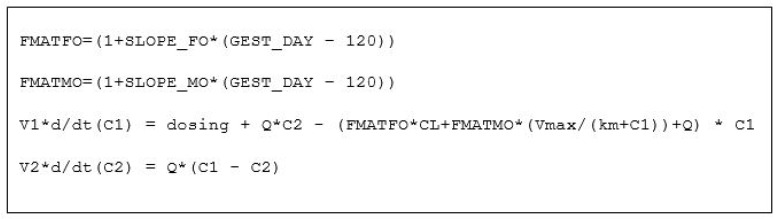
The equations describing the pharmacokinetics of rEPO in central (C1) and peripheral (C2) compartments. Input is defined by the rEPO input rate (dosing). Distribution is defined by central (V1) and peripheral (V2) volumes with inter-compartmental clearance (Q). Elimination is a combination of first-order (CL) and mixed-order (Vmax, km) elimination processes. Maturation of elimination is determined by first-order (SLOPE_FO) and mixed-order (SLOPE_MO) factors determined by a linear function of gestation day (GEST_DAY). Differential equations are indicated by d/dt.

**Figure 3:**
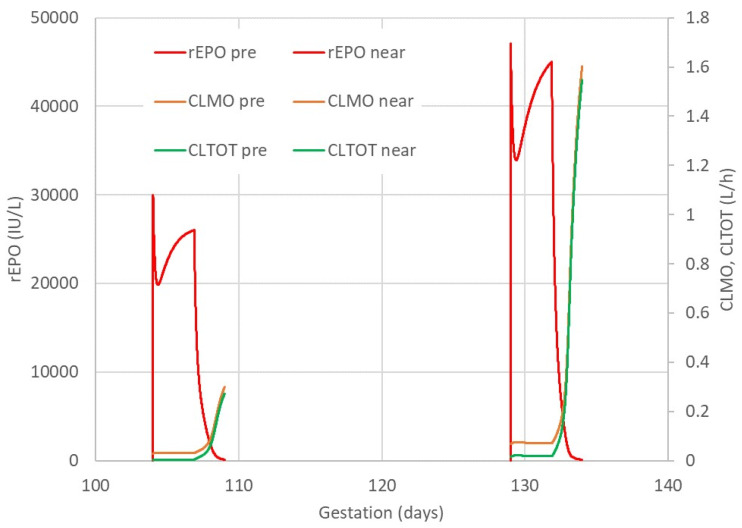
Pharmacokinetic model with combined first-order and mixed-order elimination using predicted growth in weight for size and gestation day for maturation. Predicted time course of plasma concentrations of rEPO, mixed-order clearance (CLMO) and total clearance (CLTOT = CLMO + first-order CL) following a loading infusion over 5 min then an infusion over 69 h in typical preterm and near-term fetal sheep.

**Figure 4:**
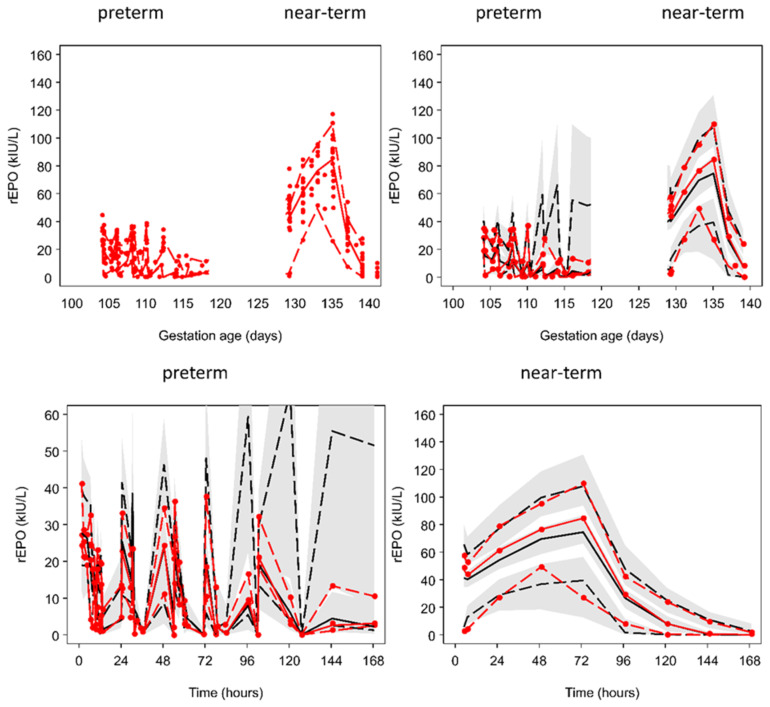
Final pharmacokinetic model assuming combined first-order and mixed-order elimination using estimated fetal weight for size scaling. Upper left hand plot is a scatterplot of all the observations. Upper right hand plot and lower plots are visual predictive checks. The solid red line represents the median observed rEPO concentration and the dashed red lines are the 5th and 90th percentiles. The solid black line represents the median predicted rEPO concentration and the dashed black lines are the 5th and 90th percentiles. The shaded areas are 95% confidence intervals for the prediction percentiles.

The observed rEPO plasma concentrations were well described by a model assuming that elimination was a combination of first-order and mixed-order elimination processes. Differences in body weight and gestation age were accounted for by using theory based allometry for distribution and elimination, and a maturation function for elimination (Figure 2). Figure 3 shows the predicted time course of rEPO in the central compartment (C1) and the accompanying time course of the clearance processes that determine the elimination of rEPO. A visual predictive check (VPC) of the observed and predicted percentiles of rEPO concentrations as a function of gestation day and of time after the first rEPO dose in preterm and near-term groups is shown in Figure 4. The predicted and observed concentrations distribution are comparable. Because a loading dose was accidentally omitted in one fetal sheep, the lower percentiles of the observed and model predicted values are lower at earlier times than they would have been if the loading dose had been given. The parameter estimates and bootstrap confidence intervals for the rEPO pharmacokinetic model are shown in Table 1.

The model described here was used to investigate whether interventions such as global asphyxia, cerebral ischemia, and hypothermia might be associated with differences in rEPO pharmacokinetics. Because of the imbalanced design of interventions, this exploratory analysis assumed that interventions have independent and additive effects. A non-parametric bootstrap of the final pharmacokinetic model was used to estimate 95% confidence intervals for the size of each intervention effect relative to the five near-term and one preterm fetal sheep that were not exposed to any of these interventions. Differences between near-term and preterm fetal sheep were assumed to be adequately described by size and maturation. The only significant difference in any of the six rEPO pharmacokinetic parameters describing disposition after an intravenous dose was associated with exposure to 30 min cerebral ischemia. There was a significant increase in CL and Q in fetal sheep exposed to cerebral ischemia (Table 2). These results should be treated with caution because the study was not powered a priori to detect the observed group differences. There was no detectable effect of the subsequent 69 h of hypothermia in this group.

**Figure 5:**
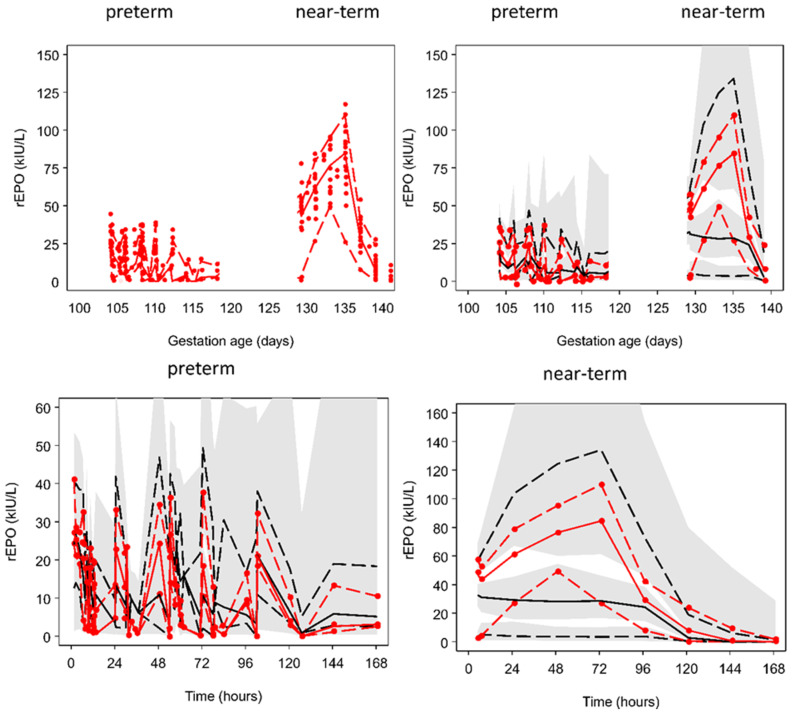
Pharmacokinetic model assuming first-order elimination and elimination by binding to EPO receptors (EPO-R) [18,23] using estimated fetal weight for size scaling. The upper left hand plot is a scatterplot of all the observations. The upper right hand plot and lower plots are visual predictive checks. The solid red line represents the median observed rEPO concentration and the dashed red lines are the 5th and 90th percentiles. The solid black line represents the median predicted rEPO concentration and the dashed black lines are the 5th and 90th percentiles. The shaded areas are 95% confidence intervals for the prediction percentiles.

The previously described model in human neonates proposed that rEPO elimination was in part explained by binding to EPO-R [18,23]. However, this model did not adequately describe the pharmacokinetics of rEPO in preterm and near-term fetal sheep. A visual predictive check (VPC) shows that the predicted concentrations do not follow the time course of observed concentrations during a prolonged rEPO infusion (Figure 5). 

### 2.4. Fetal Hematological Variables

There were no significant differences in hemoglobin concentration or hematocrit between the vehicle and rEPO treatment groups at any time during and after rEPO treatment in both preterm and near-term fetuses (Supplementary Materials Table S2). 

### 2.5. Post-Mortem Findings

**Figure 6:**
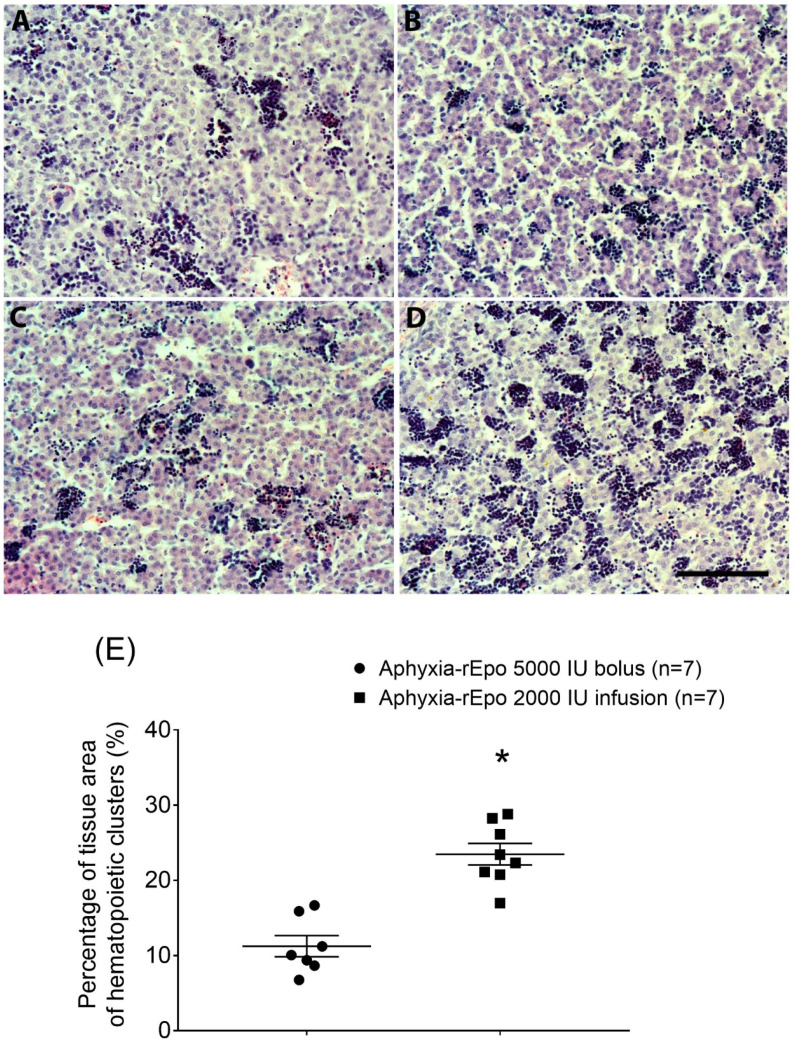
Representative photomicrographs of hematoxylin and eosin-stained liver sections in the preterm sham-asphyxia (**A**), asphyxia-vehicle (**B**), asphyxia-rEPO bolus 5000 IU (**C**) and asphyxia-rEPO infusion 2000 IU (**D**) fetal sheep groups at 7 days after asphyxia. Scale bar is 50 µm. Percentage of the area of hematopoietic cell clusters in the peri-venous area on a liver tissue section imaged at 20 X magnification (**E**) in the asphyxia-rEPO bolus 5000 IU (*n* = 7) and asphyxia-rEPO 2000 IU infusion (*n* = 7) groups at day 7 after asphyxia. Data are the average ± SEM. Figure symbol * *p* < 0.05.

There was no significant difference in post-mortem fetal body weight between the rEPO- and vehicle-treated groups (Supplementary Materials Table S3). The preterm asphyxia-rEPO 5000 IU infusion and asphyxia-rEPO 2000 IU infusion groups showed a significant increase in liver weight compared with the sham-asphyxia groups (*p* < 0.05) (Supplementary Materials Table S3). There were no macroscopic differences in hepatic structure among the preterm sham-asphyxia, asphyxia-saline and asphyxia-rEPO bolus 5000 IU fetuses (Figure 6). By contrast, there was an increase in hematopoietic clusters in the asphyxia-rEPO infusion 2000 IU group (Figure 6). Quantitative assessment showed a significantly higher percentage of the liver tissue area occupied by hematopoietic cells in the asphyxia-rEPO infusion 2000 IU group than in the asphyxia-rEPO bolus group (*p* < 0.05) (Figure 6). Fetal liver tissue from the preterm asphyxia rEPO 5000 IU infusion group was not collected for histological assessment.

## 3. Discussion

Describing the pharmacokinetics of a drug during gestation is challenging because of rapid growth in size and maturation of organ function during fetal life. For this analysis, we used established principles of size scaling and maturation developed by observing humans across a wide spectrum of age and weight [24,25,26,27]. Using these principles, the pharmacokinetics of rEPO in fetal sheep during gestation demonstrate that distribution is described by two compartment volumes, and elimination of rEPO can be described by a combination of first-order elimination with constant clearance and a saturable, mixed-order elimination process with clearance changing with concentration. Accounting for maturation of elimination processes substantially improved the fit compared with models involving the time course of rEPO exposure.

### 3.1. Body Size and rEPO Pharmacokinetics

Distribution and elimination processes necessarily increased during development and are strongly linked to body mass. We have used theory based allometric scaling of estimated fetal weight to account for size related changes in pharmacokinetic parameters. The estimated initial fetal weight per day was as expected lower in the preterm compared with the near-term group but there was no detectable difference in the slope between the two groups. The estimated slope (0.0716 kg/day) was similar to that estimated slope (0.0961 kg/day) from 88 post-mortem weights obtained in our laboratory from 100 to 136 gestation days. 

### 3.2. Gestation Age and rEPO Pharmacokinetics

A pharmacokinetic model assuming a linear increase in both linear and mixed-order rEPO elimination with increasing gestation age best described the observed concentrations in fetal sheep. This increase in distribution and elimination with increasing gestation age is consistent with the increase in fetal size and maturation of the elimination processes [26]. The increase in body weight and increase in body function determining elimination are linked. Because clearance processes increase non-linearly with weight, it is possible to distinguish these separate influences on elimination. The maturation effect has been described by a linear model over a short period of gestation but a non-linear function is clearly required to explain the full process from conception to birth and beyond [28]. Maturation of the first-order clearance had a slope of 1.94%/day in the present study, which would indicate a 31% increase between a typical preterm fetus and a typical near-term fetus (16 gestation days). The corresponding mixed-order clearance had a slope of 3.96%/day and an increase of 61%. Because the mixed-order process is essentially saturated during rEPO treatment (Figure 3), the effect of maturation on rEPO elimination is largely attributable to first-order clearance. To maintain a similar exposure level, higher infusion rates of rEPO would be needed during prolonged treatment. 

In addition to the increase in fetal size over the course of gestation, functional organ maturation during gestation contributes to progressive increase in elimination during fetal development [26]. There is evidence for changes in the expression of hepatic enzymes during gestation [29,30]. Similarly, there is an increase in renal elimination with fetal development [31]. Notably, previous studies in adult sheep and rats have shown that hepatic and renal clearance are not major contributors to in vivo rEPO elimination [32,33]. In adult humans with liver cirrhosis, normal metabolism of rEPO was maintained [34]. EPO receptor-mediated internalization and lysosomal degradation are thought to play an important role in the non-linear elimination of rEPO [35,36,37]. Studies in adult and newborn sheep showed that rEPO elimination is modified by experimentally induced changes in the EPO-R pool size. For example, bone marrow ablation induced with busulfan treatment reduced rEPO clearance in adult sheep [38], and an increase in EPO-R mRNA expression after phlebotomy-induced anemia in newborn lambs was associated with increased rEPO clearance [39]. Therefore, a change in EPO-R expression during gestation could potentially contribute to increased rEPO elimination. A widespread distribution of EPO-R expression in hematopoietic and non-hematopoietic tissues was seen during fetal development in humans [40]. In fetal sheep, there were development-related tissue-specific changes in EPO-R mRNA expression during gestation [41].

We observed higher rEPO plasma concentrations in near-term fetal sheep than preterm fetuses (Figure 1). It is likely that this was related to the higher overall per kg dose of rEPO in the near-term fetuses. Because it is not feasible to weigh fetuses in utero, rEPO doses used for these studies were calculated based on estimated fetal weights at 0.7 and 0.87 gestation using historical data from our laboratory. After standardizing per kg using measured post-mortem weights, the doses were on average 1.26-fold higher in the near-term group than in the preterm groups. Therefore, the higher plasma concentration in the near-term group may have resulted in further saturation of the non-linear elimination pathway. The good fit of observed concentrations in both preterm and near-term groups using a mixed-order elimination process in addition to a first-order mechanism supports this explanation (Figure 4).

### 3.3. The Effect of Hypoxia-Ischemia on rEPO Pharmacokinetics

Studies in full-term neonates have shown that exposure to asphyxia at birth can reduce drug clearance [42,43]. Impaired drug elimination in asphyxiated newborns may be attributed to the associated hypoxia affecting hepatic and renal function [42]. One limitation of the present study is that only one preterm fetus received sham-asphyxia followed by rEPO treatment, and therefore, we were unable to assess the independent effect of asphyxia on rEPO pharmacokinetic parameters. Thus, the present analysis cannot distinguish an effect of asphyxia on rEPO elimination in preterm fetal sheep from a difference between the preterm and near-term groups. We have used gestation day to describe the process of maturation of elimination, which is common to both groups, in order to assist in separating the effect of asphyxia from being classified as preterm.

Exposure to cerebral ischemia in near-term fetal sheep appeared to increase rEPO first-order clearance and inter-compartmental elimination. We also note that cerebral ischemia was associated with a decreased estimate for Vmax, which would diminish any increased elimination due to the first-order clearance. These changes were based on simultaneous estimation of 18 parameters describing the influence of three interventions in an unbalanced design of small groups. Because we have no prior expectation for changes in these parameters, we consider these to be fixed effect ‘nuisance’ parameters and we do not wish to draw any conclusion about the association with the interventions. A more plausible source of these differences would be an imbalance in unexplainable differences in fetuses assigned to the three intervention groups. It seems biologically implausible that localized cerebral ischemia would increase systemic drug elimination, although there is evidence for local changes. In P7 rats subjected to 60 min of cerebral hypoxia-ischemia, EPO-R expression was upregulated in the brain 24 h after HI [44]. Similarly, neonatal rats subjected to global asphyxia in utero (uterine artery occlusion for 60 min on E18) had increased EPO-R expression in the brain at P1 and P5 [45]. In 0.87 fetal sheep subjected to 10 min global asphyxia, EPO-R expression in the brain increased at 48 h post-asphyxia [46]. However, there is no evidence to suggest systemic changes in EPO-R expression after cerebral ischemia, and it is implausible that relatively small changes in brain EPO-R expression would considerably alter rEPO distribution and elimination. The most likely explanation is therefore that this is a chance finding in a relatively small study cohort.

### 3.4. Therapeutic Hypothermia and rEPO Pharmacokinetics

Therapeutic hypothermia treatment in term neonates with HIE often reduces drug clearance [47]. Studies in term neonates with HIE undergoing hypothermia have reported a lower clearance of rEPO compared with preterm neonates receiving prophylactic treatment with high-dose rEPO [4,13]. By contrast, in the present study, therapeutic hypothermia did not detectably alter rEPO pharmacokinetics in fetal sheep. These contrasting findings were most likely associated with the difference in core temperature. Selective head cooling in fetal sheep reduced the esophageal temperature on average by only 1.2 °C. In contrast, in full-term neonates with HIE treated with whole-body hypothermia, esophageal temperature is reduced by 3.5 °C [48]. The effects of therapeutic hypothermia on drug elimination are expected to be primarily due to reduced enzyme activity and possibly renal excretion by tubular transport [47]. Other factors such as redistribution of blood flow and changes in rates of tissue binding might also play a role [47]. The effect of therapeutic-hypothermia on rEPO receptor-mediated elimination has not been directly examined. The findings from the present study indicate that changes in rEPO elimination may not be detectable with small reductions in body temperature, and so the degree of hypothermia in the present studies did not detectably increase rEPO exposure. It is improbable that a moderate increase in exposure to rEPO mediated by hypothermia would have materially improved the additive neuroprotective effect, particularly given that studies in neonatal rats have shown neuroprotection with treatment using a wide range of rEPO doses [5,10,49]. We have previously reported, in near-term fetal sheep subjected to 30 min of cerebral ischemia, that the protocol reported here of an rEPO continuous infusion from 3 to 72 h independently attenuated neuronal loss [7]. We found similar suppression of microglia and apoptosis with therapeutic hypothermia and rEPO, suggesting the lack of additive benefit was mediated by overlap in the mechanisms of protection.

### 3.5. rEPO Treatment-Associated Changes in rEPO Elimination

There is both direct and indirect evidence for rEPO-mediated induction of EPO-R expression. In mice inoculated with EPO-R positive human colon adenocarcinoma cells, treatment with rEPO (600 IU/Kg, 2 times a week) was associated with an increase in EPO-R expression in tumor cells [50]. Similarly, a study in adult sheep using tracer interaction methodology and receptor-based recirculation model showed that EPO induced receptor upregulation of EPO-R contributes to rEPO elimination [51]. Therefore, in the present study, we tested a pharmacokinetic model examining rEPO exposure-induced changes in Vmax and rEPO elimination. 

The EPO-R binding model proposed by D’Cunha et al. was rejected on grounds of both log-likelihood (Supplementary Table S4) and graphical evaluation using VPCs. Two further models were tested involving induction of Vmax with exposure to rEPO. The first used a turnover model with an increase in Vmax formation rate driven by plasma concentration of rEPO. The second assumed a direct action of rEPO on Vmax delayed by distribution to an effect compartment. Both of the Vmax induction models substantially improved the log-likelihood and VPCs, but the predictions for the effect on Vmax were implausible, with an abrupt increase in Vmax after a delay of several days. The log-likelihood for the Vmax induction model was substantially less explanatory than maturation of elimination (Supplementary Tables S5–S7). We conclude that there is no evidence that the parameters determining the non-linear elimination of rEPO are changed by rEPO exposure.

A limitation of the present study is that EPO-R expression was not directly quantified. Interestingly, we observed an increase in liver weight in the preterm fetuses treated with prolonged continuous infusion. Further, treatment with a loading dose of 2000 IU followed by a continuous infusion at 520 IU/h for 66 h in preterm fetal sheep was associated with an increase in the percentage of liver tissue occupied by hematopoietic cells. These data suggest that treatment with prolonged rEPO infusion induced an increase in hepatic hematopoiesis in preterm fetal sheep. In fetal sheep, the liver is the primary site for hematopoiesis until gestation day 130 [52]. Erythroid progenitor cells express a high density of EPO-R [18]. This rEPO infusion treatment-mediated increase in hematopoietic clusters in the preterm fetal liver could lead to increased red blood cell production. However, rEPO treatment was not associated with a change in hemoglobin and hematocrit in these studies. Potentially a longer period of recovery might be required to assess these variables. Therefore, changes in red blood cell production are very unlikely to have contributed to neuroprotection in these studies. In summary, the pharmacokinetic analysis shows that the increase in hematopoiesis, and presumptive associated increase in EPO-R expression after prolonged rEPO exposure, does not have a detectable effect on rEPO elimination. 

### 3.6. Higher rEPO Dose Required in Fetal Sheep Compared to Human Neonates

These studies showed that a bolus dose of 5000 IU/kg in preterm fetal sheep achieves an average (AUC_0–48_) of 110,998 ± 7027 IU/L*h for rEPO plasma concentration. By comparison, treatment with a bolus dose of 1000 IU/kg in human preterm neonates achieves AUC_0–∞_ 81,498 ± 7067 IU/L*h [12]. The difference in dose required to achieve comparable rEPO exposure might be related in part to the effect of the in utero environment on drug elimination processes [53]. The estimates of pharmacokinetic parameters in fetal sheep and human neonates cannot be directly compared because the study in preterm neonates used a non-compartmental analysis method that assumes first-order elimination, whereas our study in fetal sheep used a pharmacokinetic model with both first-order and mixed-order process to describe rEPO elimination. 

Studies in fetal sheep have shown that rEPO does not cross the ovine placenta [54,55]. However, EPO-R mRNA is expressed in the ovine placenta throughout gestation in sheep, but at much lower levels than in the kidneys and liver [41]. Because of differences in size and maturation between fetal sheep and preterm neonates, it is very likely that there are differences in the total size of the EPO-R pool. Compared with the high levels of hematopoiesis in utero, and the expansion of erythroid tissue in the present study after rEPO, preterm neonates have reduced hematopoiesis, leading to physiological anemia in the post-natal period [56].

### 3.7. Conclusions

In conclusion, the present study demonstrates that the plasma concentrations of rEPO in fetal sheep after treatment with high-dose rEPO continuous infusion or boluses were best described by a pharmacokinetic model with first-order and mixed-order elimination, accounting for an increase in elimination due both to a change in size and in maturation associated with gestation age. These findings suggest that to maintain target exposure levels, the dose of rEPO may need to be increased to match growth and development both in utero and after birth. These results will be fundamental for future studies examining rEPO treatment for neuroprotection. 

## 4. Methods

All procedures were approved by the Animal Ethics Committee of the University of Auckland (R1359, March 2014–February 2017, R1942, March 2017–2021) and carried out in accordance with the Code of Animal ethical conduct established by the Ministry of Primary Industries of New Zealand for the use of animals for teaching and research. The experiments are reported in accordance with the ARRIVE guidelines for reporting animal research [57]. Romney–Suffolk cross fetal sheep were instrumented on gestation day 99 (preterm, *n* = 36) or at day 125 (near-term, *n* = 25) (full term ~147 days). All the surgical procedures were performed using aseptic techniques, as previously described [7,19,20,21]. Fetal instrumentation at both gestations included brachial artery catheters for blood pressure measurement and preductal blood sample collection, and brachial vein catheters for drug infusion. 

For studies in preterm fetal sheep, an inflatable silicone occluder (OC16HD, 16 mm, In Vivo Metric, Healdsburg, CA, USA) was loosely placed around the umbilical cord to allow post-surgical occlusion of the umbilical cord to induce fetal asphyxia. For the studies in near-term fetuses, the vertebral-occipital anastomoses were ligated, and inflatable silicone occluders were placed bilaterally around the carotid arteries for post-surgical carotid artery occlusion to induce global cerebral ischemia. A thermistor (Replacement Parts Industries, Inc., Simi Valley, CA, USA) was placed in the esophagus at the level of heart for measurement of core temperature, and a second thermistor was placed over the dura 30 mm anterior to bregma for measurement of extradural temperature. A cooling coil constructed with silicone tubing (3 × 6 mm, Degania Silicone Ltd, Degania Bet, Israel) was secured to the fetal head for inducing therapeutic hypothermia. Following surgery, fetuses were continuously monitored throughout the duration of experiments. 

Animals were housed together in the same room, in separate metabolic cages with access to concentrated pelleted food and water ad libitum. Rooms were temperature controlled (16 ± 1 °C, humidity 50 ± 10%) with a 12:12 h light:dark cycle. A period of 4–5 days of recovery was allowed before the commencement of experiments. Antibiotics were given *i.v.* to the ewe each day for 4 days—600 mg benzylpenicillin sodium (Novartis, Auckland, New Zealand) and 80 mg gentamicin (Pfizer, Auckland, New Zealand). Fetal vascular catheters were continuously infused with heparinized saline (20 U/mL at 0.2 mL/h) to maintain patency. The fetal condition was assessed via recordings of all fetal physiological variables, and daily arterial samples were drawn to monitor pH and blood gases (ABL800 Flex analyzer, Radiometer, Auckland, New Zealand), and glucose and lactate (YSI 2300 Analyzer, YSI Ltd., Yellow Springs, OH, USA), as previously described [20,21]. 

### 4.1. Studies in Preterm Fetal Sheep

In preterm fetal sheep (0.7 gestation), global HI was induced with complete occlusion of the umbilical cord for 25 min, as previously described [19,21]. Initially, pilot studies were conducted to assess the independent effect of exposure to global HI on rEPO pharmacokinetics. On gestation day 104 ± 1 (0.7 gestation, full term 147 days), preterm fetal sheep were subjected to sham-asphyxia (*n* = 1) or 25 min umbilical cord occlusion (UCO) (*n* = 3) followed by treatment with rEPO (loading dose 3000 IU, followed by a continuous infusion at 500 IU/h (i.v.) from 6 to 168 h post-UCO). 

**Figure 7:**
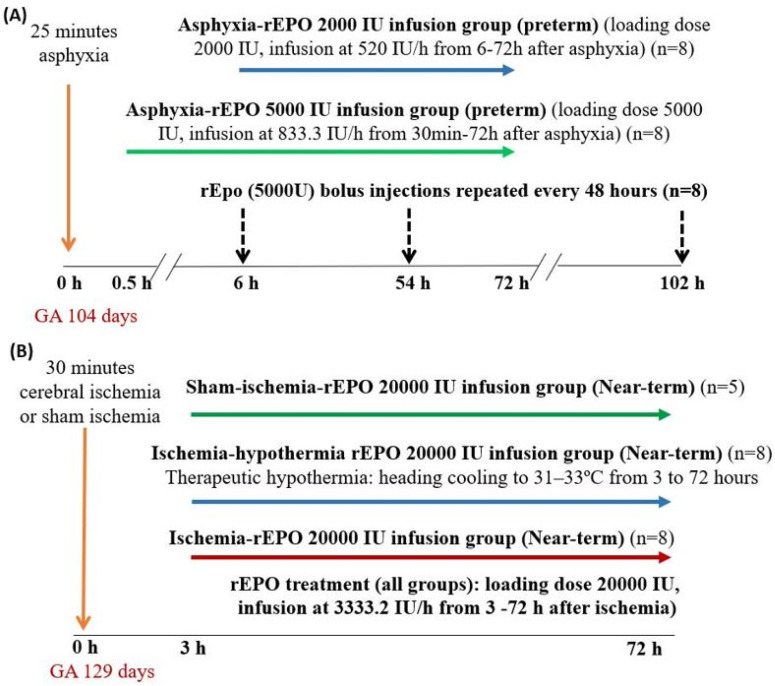
Summary of rEPO treatment regimens in preterm (Panel **A**) and near-term (Panel **B**) fetal sheep studies.

For the subsequent studies, fetuses were subjected to 25 min of UCO, followed by randomization to one of the rEPO treatment protocols (Figure 7). 

Protocol 1: rEPO was administered as a rapid loading infusion of 5000 IU over 1 min, followed by infusion at 833.3 IU/h from 30 min to 72 h post-HI (*n* = 8).

Protocol 2: rEPO was administered as a rapid loading infusion of 2000 IU over 2 min, followed by a continuous infusion at 520 IU/h from 6 to 72 h post-HI (*n* = 8).

Protocol 3: Single rapid infusion (bolus dose) of 5000 IU over 2 min starting 6 h post-UCO, then repeated every 48 h for three doses (*n* = 8).

These doses were based on estimated fetal weight of 1 kg at gestation day 104, based on the historical data in our laboratory. The dose of rEPO used in these studies is higher than that used in clinical trials in preterm infants (1000–3000 IU/Kg) because a greater dose of rEPO is required in preterm fetal sheep to achieve comparable plasma exposure levels to that reported in preterm infants, as shown above. In the 2000 and 5000 IU infusion groups, the plasma concentrations of rEPO were on average maintained higher than 10,000 IU/L, which was the threshold plasma concentration for neuroprotective dose in neonatal rats [10,11]. Furthermore, treatment with a bolus dose of 5000 IU rEPO in fetal sheep on gestation day 95 significantly increased rEPO concentrations in the cerebrospinal fluid at 3 h after the injection [58]. 

Post-mortems were conducted at 72 h (asphyxia-rEPO 5000 IU infusion group) or 7 days (rEPO 5000 IU bolus or rEPO 2000 IU infusion groups) post-UCO. Ewes and fetuses were killed with an overdose of sodium pentobarbitone (9 g intravenous to the ewe; Pentobarb 300; Chemstock, Christchurch, New Zealand). At post-mortem, fetal organs were weighed and the patency of venous catheters was examined with dye infusion. For asphyxia-rEPO 5000 IU bolus and asphyxia-rEPO 2000 IU infusion groups, the fetal liver tissue was collected at post-mortem for histological assessment. 

### 4.2. Studies in Near-Term Fetal Sheep

In near-term fetal sheep (0.87 gestation), HI brain injury was induced by reversible bilateral carotid artery occlusion for 30 min. On gestation day 129 ± 1 days (0.87 gestation, full term 147 days), fetuses were randomly assigned one of the following groups (Figure 7), as described previously [7].

Protocol 1: Sham-ischemia followed by rEPO treatment (*n* = 5).

Protocol 2: Ischemia-vehicle treatment (*n* = 4).

Protocol 3: Ischemia-rEPO treatment (*n* = 8).

Protocol 4: Ischemia followed by concurrent treatment with rEPO and therapeutic hypothermia (extradural temperature lowered to 31 to 33 °C (*n* = 8)).

rEPO treatment in all the groups was administered as intravenous (i.v.) rapid infusion of a loading dose of 20,000 IU over 5 min, followed by a continuous infusion at 3333.2 IU/h over 69 h starting from 3 h after ischemia, based on the estimate of the fetal weight of 4 kg at gestation day 129, based on the historical data in our laboratory. Therapeutic hypothermia was performed by circulating cold water through the coil over the fetal scalp using a pump (TX150 Heating circulator, Grant Instruments Ltd., Cambridge, UK) that was placed in a cooled water bath, as described previously [7,20]. Combined treatment with hypothermia and rEPO was only included for the near-term group. Therapeutic hypothermia is the standard of care for term neonates with moderate to severe HIE and, therefore, it is important to examine whether treatment with rEPO offers additive protective effects when combined with therapeutic hypothermia. Therapeutic hypothermia is not available for preterm infants due to potential safety concerns, and therefore combined treatment with hypothermia and rEPO was not examined for preterm groups in the present study. Post-mortems in near-term groups were conducted at seven days post-ischemia. 

### 4.3. Fetal Arterial Blood Sampling and rEPO Concentration Measurement

The fetal arterial blood sampling regimen was designed for rEPO pharmacokinetic analysis. Supplementary Table S8 describes the details of the timing of fetal arterial blood sample collection for rEPO measurements. In addition, daily fetal arterial blood samples were taken to assess the fetal condition, as previously described [20,22]. Fetal arterial blood samples for rEPO concentration measurement were collected on ice in EDTA vacutainers (Onelink, Auckland, New Zealand) and immediately centrifuged at 3000 rpm for 10 min at 4 °C (Heraeus Megafuge 8R, Thermo Fisher Scientific Ltd., Auckland, New Zealand) and fetal plasma was collected and stored at −80 °C for analysis. rEPO concentrations in fetal plasma were measured in duplicate using the commercial Quantikine human erythropoietin enzyme-linked immunosorbent assay (DEP00, R&D Systems, Minneapolis, MN, USA) according to manufacturer’s instructions. The assay was validated for fetal sheep plasma by running plasma samples spiked with a known concentration of rEPO (50 IU/L and 100 IU/L). Human serum, with reference rEPO concentrations (Control Erythropoietin plasma 1 and 3, R&D Systems), and negative controls were included in every assay to evaluate the assay precision. The standard series ranged from 2.5 to 200. The lower limit of quantification of the assay was 2.5 IU/L, and the lower limit of detection was 0.6 IU/L. The samples were run with the dilution range of 1:10 to 1:800, or undiluted when required. The samples with measured concentration below the reported lower limit of quantification were treated as missing in the pharmacokinetic analysis. The average intra-assay and inter-assay coefficients of variability (CV) were 2.7% and 8.1%, respectively. Consistent with this, an inter-assay CV range of 2.4% to 7.8% is reported by the manufacturer. Previously, a study in term human infants reported an inter-assay CV < 11% using the same commercial assay kit [13].

### 4.4. Pharmacokinetic Analysis

The time course of rEPO concentrations was described with a joint pharmacokinetic model of data from preterm and near-term fetal sheep. The rEPO input was defined by the administered dose and duration of a constant rate infusion for all doses. The model selection process is described in the Supplementary material. Distribution was described by two compartments, central and peripheral, with an apparent volume of distribution for each compartment (V1 and V2) and an inter-compartmental clearance (Q). Elimination was assumed to occur from the central compartment. Two elimination processes were identifiable—a first-order process and a mixed-order process. The first-order elimination process was defined by clearance (CL). The mixed-order elimination process was defined by two parameters—Vmax, the maximum elimination capacity, and km, the rEPO central compartment concentration when the rate of elimination of rEPO is 50% of Vmax. 

The fetal weight data were obtained at post-mortem. Based on historical data in our laboratory, it is known that post-mortem weight increases with gestation age. Because weight is linked through allometric theory to pharmacokinetic parameters, it was possible to estimate the initial in utero weight under the constraint of a linear relationship between weight and gestation age, leading to the post-mortem weight observation. A mixed-effects linear model was used to describe the post-mortem weight observations and impute in utero weight [59]. Random effects were estimated to describe the between-fetus variability in initial weight and slope parameters, along with an additive residual error (Supplementary Table S5). Supplementary Figure S3 shows a visual predictive check (VPC) of the observed and predicted percentiles of rEPO concentrations using a pharmacokinetic model assuming combined first-order and mixed-order elimination using post-mortem weight for size scaling.

A mixed-effects model was used to define between-sheep variability in pharmacokinetic parameters and random unexplained variability in the measured concentrations [24,25]. Predictable (fixed effect) sources of variability were investigated based on predicted fetal weight at each dose and concentration measurement, preterm or term group, asphyxia, ischemia and hypothermia.

As described above, it has been previously reported that rEPO elimination is linked to the extent of binding with EPO-R. In the preterm asphyxia-rEPO infusion 2000 IU group, there was evidence of increased liver weight and hematopoietic cell mass in the liver at 7 days after the start of rEPO treatment, which may be the primary mechanism of rEPO clearance mediated by increased EPO-R. A model was developed to describe an induction effect of rEPO exposure on a tissue assumed to be EPO-R and hereafter described as ERmass (Supplementary Table S5).

In this model, the effect of rEPO on ERmass induction takes time, with a delay described by a mean transit time (MTT) using a series of transit compartments. The output of the transit chain is an effect concentration (Ce). ERmass increase is driven by Ce using a sigmoid Emax model with maximum effect EmaxVM, sensitivity C50VM, defined as Ce at 50% of EmaxVM, and steepness HillVM. The fold increase in rEPO elimination capacity, VmaxT, changes with rEPO exposure and thus with time and is assumed to be proportional to ERmass. 

Parameter estimation was performed using NONMEM 7.4.4 [60] and Wings for NONMEM [61]. The model evaluation used visual predictive checks [62,63] and non-parametric bootstrap statistics describing the parameter estimates [64,65].

### 4.5. Histology

The liver tissue was immersion-fixed in 10% phosphate-buffered formalin (Global Science, Auckland, New Zealand). The fixed tissue was processed using an automated enclosed tissue processor (Leica APS 300S, Bio-Strategy Ltd., Auckland, New Zealand), and then embedded in paraffin wax (Paraplast^®^ Regular, Sigma-Aldrich, St. Louis, MO, USA). The 10 µm transverse sections were cut using a microtome (Leica Jung RM2035, Leica Microsystems Ltd., Albany, New Zealand), and mounted on poly-L-lysine (Sigma-Aldrich)-coated slides. For histological assessment, sections were deparaffinized in xylene, rehydrated in graded series of ethanol, and then washed in phosphate-buffered saline (0.1 mol/L). Hematoxylin and eosin staining was performed using standard procedures, and then the tissue was dehydrated in increasing concentrations of alcohol and mounted. Sections were imaged using light microscopy (Nikon Eclipse 80i, Scitech Ltd., Preston, Australia) at 20x magnification (image size 512 µm × 384 µm), and hematopoietic cells and hepatocytes were identified by morphological features. Quantitative assessment was performed by measuring the area of the tissue occupied by hematopoietic cell clusters on the 20X magnification images using Image J. An average of five fields were taken from each section. 

### 4.6. Statistical Analysis

The data for fetal hematology, post-mortem weights and liver hematopoietic cluster area were evaluated by analysis of variance (ANOVA, SPSS version 23, SPSS Inc., Chicago, IL, USA). Where a significant overall effect of group was found, between-group comparisons were performed by univariate analysis with LSD pair-wise comparisons. Statistical significance was accepted as *p* < 0.05. Data are presented as the average ± SEM. 

## Figures and Tables

**Table 1:** Parameter estimates for rEPO pharmacokinetics, maturation and gestation weight. Original and non-parametric bootstrap (200 replicates).

Parameter	Description	Units	Original	Bootstrap Average	2.5% ile	97.5% ile	RSE
CL	rEPO first-order clearance	L/h/70 kg	0.471	0.506	0.399	0.663	15.6%
Vmax	rEPO elimination capacity	IU/h/70kg	4830	5342	2501	11,130	49.6%
Km	rEPO Km	IU/L	441	560	157	1394	103.5%
V1	rEPO central volume	L/70 kg	7.67	7.98	6.49	9.98	11.3%
Q	rEPO distribution clearance	L/h/70 kg	0.379	0.383	0.229	0.547	24.7%
V2	rEPO peripheral volume	L/70 kg	13.4	13.2	8.1	17.7	18.8%
SLOPE FO	Slope of maturation for first-order elimination	1/day	0.0194	0.0156	−0.0111	0.0363	81.9%
SLOPE MO	Slope of maturation for mixed-order elimination	1/day	0.0396	0.0371	0.0127	0.0530	34.9%
WT0 preterm	Initial weight in preterm	kg	1.50	1.49	1.25	1.73	8.0%
WT0 near-term	Initial weight near-term	kg	3.82	3.81	3.37	4.24	5.9%
WT slope	Weight slope	kg/d	0.072	0.075	0.021	0.132	40.0%
PPV CL	PPV Clearance	.	0.023	0.028	0.000	0.139	134.7%
PPV VMAX	PPV Vmax	.	0.520	0.489	0.288	0.683	22.8%
PPV km	PPV km	.	1.08	1.04	0.583	1.46	20.5%
PPV V1	PPV V1	.	0.211	0.151	0.00200	0.300	61.2%
PPV WT0	PPV initial weight	.	0.046	0.081	0.000	0.191	80.5%
PPV WTslope	PPV slope of weight	.	0.727	0.597	0.007	1.263	75.3%
R12	Correlation CL with Vmax	.	0.804	0.496	−0.976	1.00	127.6%
R13	Correlation CL with km	.	0.992	0.693	−0.760	1.00	68.4%
R23	Correlation Vmax with km	.	0.872	0.834	0.572	0.986	28.3%
RUV PROP rEPO	Proportional residual error for rEPO	.	0.211	0.203	0.160	0.244	11.0%
RUV ADD rEPO	Additive residual error for rEPO	IU/L	5.08	4.92	2.56	7.96	28.0%
RUV ADD WT	Additive residual error for post-mortem weight		0.367	0.368	0.217	0.489	19.6%

PPV = population parameter variability (sqrt(NONMEM omega)); R = correlation co-efficient; RUV = residual unidentified variability; RSE = bootstrap relative standard error (100× sqrt(bootstrap SD)/bootstrap average); Km = Michaelis constant. The “.” Character indicates there are no units for this parameter.

**Table 2:** Parameter estimates for global asphyxia, cerebral ischemia and hypothermia interventions associated with rEPO pharmacokinetic disposition parameters. Parameters estimated jointly with those in Table 1. Original and non-parametric bootstrap (200 replicates).

Parameter	Description	Original	Bootstrap Average	2.5% ile	97.5% ile	RSE
FV1 ASP	Asphyxia on V1	1.030	0.982	0.773	1.210	12.0%
FV2 ASP	Asphyxia on V2	0.419	0.480	0.174	0.886	36.6%
FQ ASP	Asphyxia on Q	0.570	0.646	0.157	1.242	41.7%
FCL ASP	Asphyxia on CL	1.390	1.266	0.385	2.169	35.4%
FVM ASP	Asphyxia on Vmax	2.040	2.146	0.723	4.270	45.4%
FKM ASP	Asphyxia on km	2.890	3.774	0.295	19.800	110.3%
FV1 ISC	Ischemia on V1	0.872	0.862	0.648	1.080	13.5%
FV2 ISC	Ischemia on V2	1.200	1.174	0.889	1.685	17.9%
FQ ISC	Ischemia on Q	1.790	1.749 *	1.107	2.651	23.8%
FCL ISC	Ischemia on CL	1.510	1.463 *	1.110	1.942	14.7%
FVM ISC	Ischemia on Vmax	0.505	0.543	0.171	0.984	43.9%
FKM ISC	Ischemia on km	0.850	0.967	0.224	2.888	68.8%
FV1 COOL	Hypothermia on V1	1.320	1.265	0.886	2.083	25.3%
FV2 COOL	Hypothermia on V2	1.170	1.169	0.731	1.820	20.5%
FQ COOL	Hypothermia on Q	1.330	1.337	0.769	2.132	25.6%
FCL COOL	Hypothermia on CL	1.200	1.181	0.888	1.502	15.8%
FVM COOL	Hypothermia on Vmax	0.775	0.831	0.330	1.495	38.6%
FKM COOL	Hypothermia on km	0.884	1.026	0.233	2.875	64.0%

* =95% confidence interval does not include 1.

## Supplementary Materials

Supplementary materials can be found at https://www.mdpi.com/1422-0067/21/9/3042/s1.
