# Supplementary material for: The Effect of Size, Maturation, Global Asphyxia, Cerebral Ischemia, and Therapeutic Hypothermia on the Pharmacokinetics of High-Dose Recombinant Erythropoietin in Fetal Sheep
Source: Int J Mol Sci. 2020 Apr 25;21(9):3042. doi: 10.3390/ijms21093042 (PMC7247678; doi:10.3390/ijms21093042)
Supplement: Supplementary file 1 [file ijms-21-03042-s001.pdf]

Supplementary data

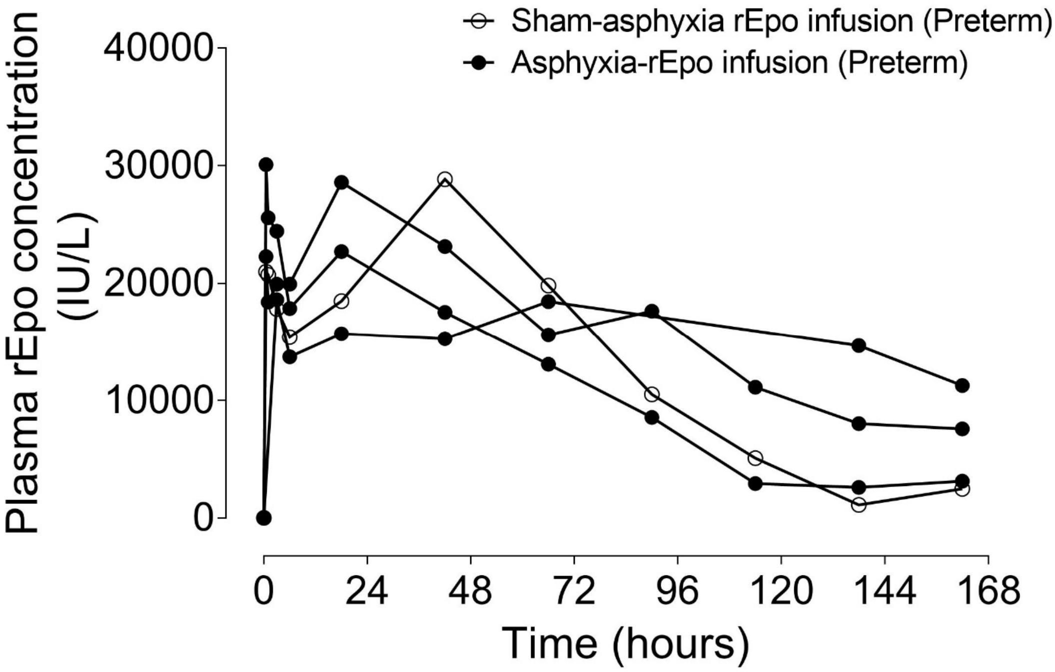

Supplementary Figure S1: Time sequence of plasma concentrations of rEPO (IU/L) in the preterm asphyxia-rEPO infusion (closed circles) (n=3) and sham-asphyxia-rEPO groups (open circles) (n=1) at 1 hour pre-rEPO, 30 minutes, 1, 3, 21, 42, 66, 90, 114, 138 and 162 hours during rEPO infusion. rEPO was administered as a loading dose of 3000 IU followed by a continuous infusion at 500 IU/h from 6 to 168 hours after asphyxia. Data show 4 individual animals.

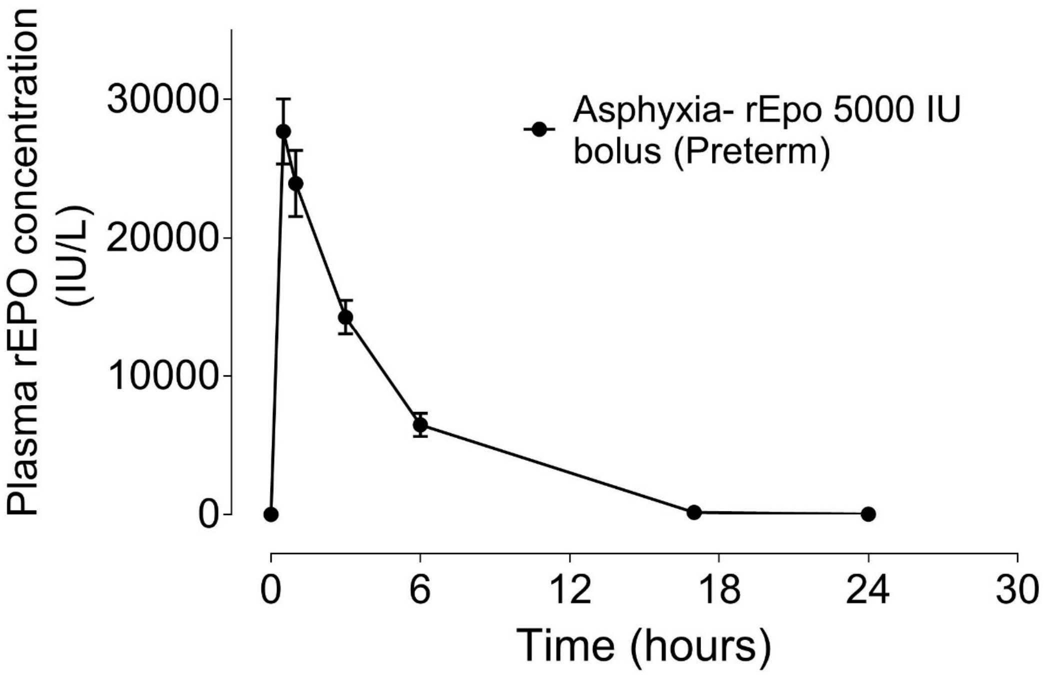

Supplementary Figure S2: Time sequence of plasma concentrations of rEPO (IU/L) in the preterm asphyxia-rEPO 5000 IU bolus group at 1-hour pre-EPO, 30 minutes, 3, 6, 17 and 24 hours after an intravenous bolus injection of rEPO. Data are presented as average ± SEM.

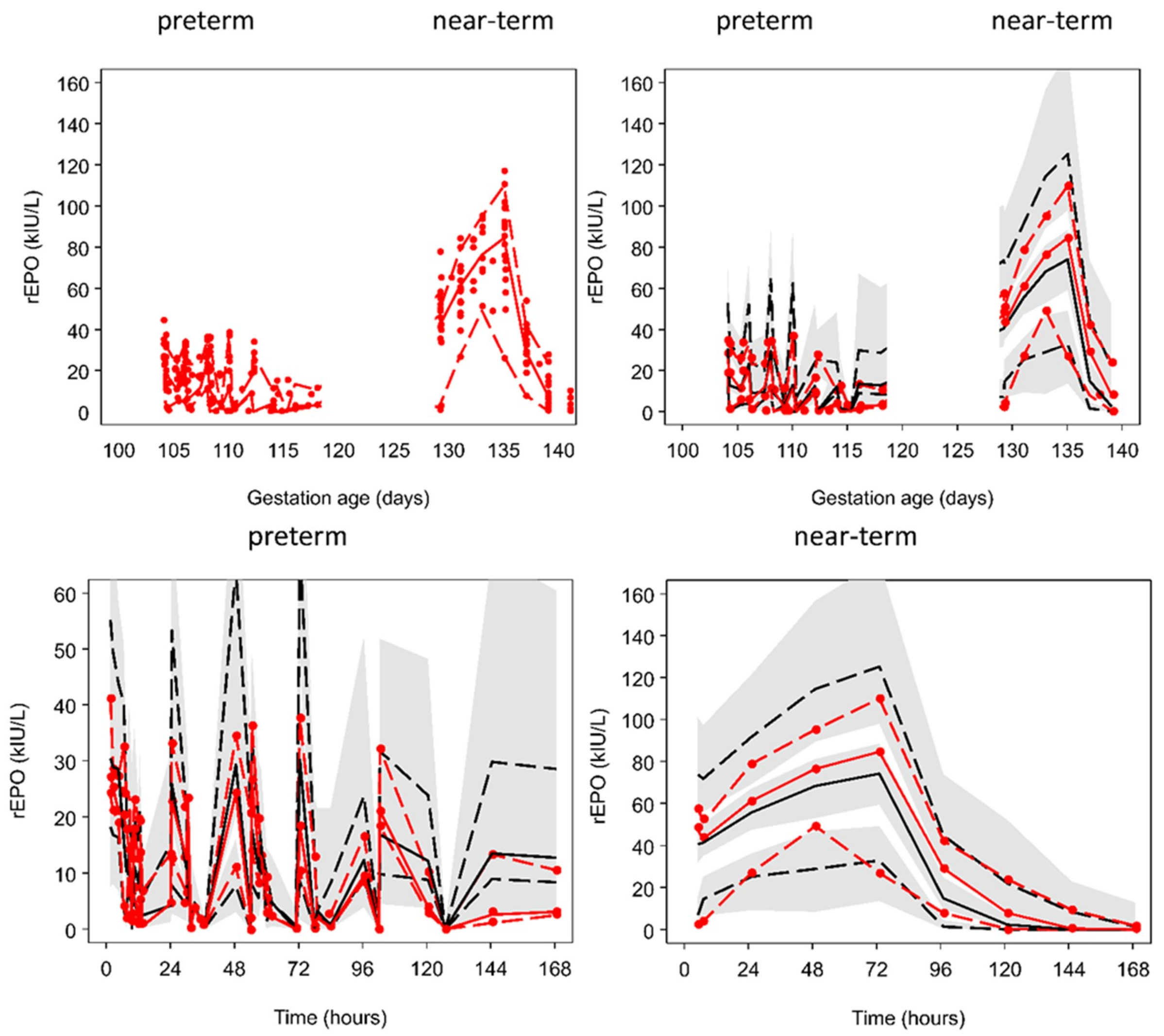

Supplementary Figure S3: Pharmacokinetic model assuming combined first-order and mixed order elimination using post-mortem weight for size scaling. The upper left hand plot is a scatterplot of all observations. The upper right hand plot and lower plots are visual predictive checks. The solid red line represents the median observed rEPO concentration, and the dashed red lines are the 5th and 90th percentiles. The solid black line represents the median predicted rEPO concentration, and the dashed black lines represent the 5th and 90th percentiles. The shaded areas are 95% confidence intervals for the prediction percentiles.

Supplementary Table S1: rEPO plasma concentrations at 24, 48 and 72 hours after HI in preterm and near-term fetal sheep treated with a loading bolus followed by prolonged continuous infusion of rEPO. Data are average ± SEM.

	**rEPO plasma concentration (IU/L)**		
	**24 hours**	**48 hours**	**72 hours**
Asphyxia-rEPO 2000 IU (Preterm)	15200.4 ± 2023.1	13072 ± 2513	6502.8 ± 2081.7
Asphyxia-rEPO 5000 IU (Preterm)	24398.4 ± 2575.1	25678.6 ± 2691.8	24910.0 ± 3566.3
Ischemia-rEPO 20000 IU (Near-term)	62161.1 ± 4094.1	77894.0 ± 4683.1	90738.7 ± 6292.8

Supplementary Table S2: Fetal hematology measurements 1 hour before, 24, 48 and 72 hours after ischemia or asphyxia in near-term and preterm fetal sheep. Data are average ± SEM.

	pre-rEPO	24 hours	48 hours	72 hours
**Hemoglobin (g/dL)**				
Sham-ischemia rEPO 20000 IU	10.3 ± 0.3	10.0 ± 0.2	10.5 ± 0.6	10.6 ± 0.4
Ischemia-vehicle	9.9 ± 0.5	9.8 ± 0.7	9.4 ± 0.6	9.7 ± 0.7
Ischemia-rEPO 20000 IU	9.9 ± 0.5	10.0 ± 0.5	9.8 ± 0.6	10.2 ± 0.6
Ischemia-hypothermia rEPO 20000 IU	10.1 ± 0.3	10.3 ± 0.4	10.1 ± 0.4	10.3 ± 0.4
Sham-asphyxia	8.6 ± 0.2	8.7 ± 0.2	8.7 ± 0.3	8.5 ± 0.2
Asphyxia-vehicle	9.4 ± 0.3	9.3 ± 0.5	9.2 ± 0.4	9.0 ± 0.3
Asphyxia-rEPO 5000 IU infusion	9.3 ± 0.5	9.5 ± 0.6	9.8 ± 0.7	10.2 ± 1.0
Asphyxia-rEPO 2000 IU infusion	9.9 ± 0.6	9.9 ± 0.5	9.6 ± 0.5	9.5 ± 0.5
Asphyxia-rEPO 5000 IU bolus	9.3 ± 0.2	9.9 ± 0.3	9.2 ± 0.3	8.9 ± 0.3
**Hematocrit (%)**				
Sham-ischemia rEPO 20000 IU	30.4 ± 0.9	29.2 ± 0.6	29.9 ± 1.6	30.9 ± 1.2
Ischemia-vehicle	29.1 ± 1.8	28.6 ± 2.0	27.8 ± 1.8	28.5 ± 2.2
Ischemia-rEPO 20000 IU	29.1 ± 1.4	29.4 ± 1.6	28.8 ± 1.8	29.9 ± 1.7
Ischemia-hypothermia 20000 IU	29.8 ± 1.0	30.4 ± 1.3	29.6 ± 1.2	30.0 ± 1.0
Sham-asphyxia	25.1 ± 0.7	25.1 ± 0.5	25.1 ± 0.7	25.0 ± 0.6
Asphyxia-vehicle	27.8 ± 0.6	28.3 ± 1.3	27.8 ± 1.0	27.3 ± 0.8
Asphyxia-rEPO 5000 IU infusion	26.8 ± 1.4	28.1 ± 2.7	28.9 ± 2.2	29.8 ± 2.9
Asphyxia-rEPO 2000 IU infusion	29.0 ± 0.8	30.8 ± 0.9	29.7 ± 1.5	29.7 ± 1.7
Asphyxia-rEPO 5000 IU bolus	29.0 ± 0.8	30.8 ± 0.9	28.6 ± 1.1	27.7 ± 0.9

Supplementary Table S3: Fetal sheep body and liver weights at post-mortem. Data are average ± SEM. Table symbols are * $P < 0.05$ versus the sham-asphyxia group.

	**Body weight (Kg)**	**Liver weight (g)**
Sham-ischemia rEPO 20000 IU infusion (Near-term)	4.9 ± 0.2	103.6 ± 11.2
Ischemia-vehicle (Near-term)	4.6 ± 0.3	113.4 ± 8.7
Ischemia-rEPO 20000 IU infusion (Near-term)	4.4 ± 0.2	136.3 ± 12.7
Ischemia-hypothermia rEPO 20000 IU infusion (Near-term)	4.7 ± 0.1	143.2 ± 15.3
Sham-asphyxia 72 hours (Preterm)	1.5 ± 0.5	64.2 ± 3.6
Asphyxia-vehicle 72 hours (Preterm)	1.7 ± 0.1	76.7 ± 3.0
Asphyxia-rEPO 5000 IU infusion 72 hours (Preterm)	1.5 ± 0.6	106.8 ± 6.9*
Sham-asphyxia 7 days (Preterm)	2.5 ± 0.4	74.5 ± 4.1
Asphyxia-vehicle 7 days (Preterm)	2.1 ± 0.1	84.5 ± 3.9
Asphyxia rEPO 2000 IU infusion 7 days (Preterm)	2.0 ± 0.1	101.3 ± 4.5*
Asphyxia rEPO 5000 IU bolus 7 days (Preterm)	2.1 ± 0.1	85.1 ± 3.0

Supplementary Material Model Selection

Model selection was guided by mechanism, parameter estimate plausibility and the improvement in NONMEM's objective function value (OFV).

In the following tables, each model is associated with a Test model name. When the Comparison model is used to compare with several Test models then a short descriptor is used for the Test model name otherwise the model number is used. The Conclusion column is based on the likelihood ratio test. The log likelihood ratio is assumed to be chi-square distributed in order to estimate a P value to reject the Test column statement. A P value < 0.01 was used to reject the null hypothesis.

The Final rEPO pharmacokinetic and estimated weight model steps and OFV changes are shown in **Supplementary Table S5** and **Supplementary Table S7**. This demonstrates that a combined mixed order (MO) and first-order (FO) elimination process is better than FO alone. The use of an estimated weight during gestation is superior to using post-mortem weight. Adjusting the 6 key pharmacokinetic parameters (CL, V1, Q, V2, Vmax and km) for differences associated with the 3 interventions (global asphyxia, cerebral ischemia, and combined hypothermia (cooling) and ischemia (CI) further improved the fit. The final model was then obtained by including a linear maturation model for FO and MO elimination based on gestation age centered at 120 days.

The fit of the base mixed order (MO) elimination model compared with an EPO-R binding elimination model is shown in **Supplementary Table S4** and **Supplementary Table S6**. The EPO-R model described by d'Cunha et al. used an allometric exponent of 0.75 to describe size associated changes in the rEPO elimination rate constant (kp). The OFV improved substantially when a theory based allometric exponent of ¾ was used to describe size associated changes in rEPO clearance (CL) and central volume (V1) and kp then calculated from CL/V1. The EPO-R elimination mechanism was still rejected in comparison with MO elimination.

Supplementary Table S4: MO compared with EPO-R elimination models: goodness of fit using NONMEM objective function value (OFV) and likelihood ratio test (chi-square)

Model Number<br>Model Identifier	Test Model	Reference Model	OFV	dOFV	df	chi-sq P
0059<br>k02_allo_linwt1add_pre---_-------_----	BaseFOMO	.	7428.569	.	.	.
0057<br>k02_allo_linwt1add_preall_EPO-R_CL_conc	0057	BaseFOMO	7747.591	319.022	4	8.53E-68
0058<br>k02_allo_linwt1add_pre---_EPO-R_kp	0058	BaseFOMO	7894.278	465.709	4	1.7E-99

Difference in OFV (dOFV) = abs(Test model – Reference model)

Supplementary Table S5: rEPO pharmacokinetic and estimated weight models: goodness of fit using NONMEM objective function value (OFV) and likelihood ratio test (chi-square)

Model Number<br>Model Identifier	Test Model	Reference Model	OFV	dOFV	df	chi-sq P
0092<br>k02_allo_linwt1add_aiHI_PCAlin_FOMO	Final	AICI	7354.075	39.27	2	2.97E-09
0095<br>k02_allo_linwt1add_aiHI_Tover_Vmax	0095	Final	7372.379	18.304	2	0.000106
0088<br>k02_allo_linwt1add_aiCI_-------_----	AICI	BaseFOMO	7393.345	35.224	18	0.008857
0059<br>k02_allo_linwt1add_pre---_-------_----	BaseFOMO	BaseFO	7428.569	539.355	5	2.5E-114
0026<br>k02_allo_PostMwt--_pre---_------_----	0026	BaseFOMO	7601.029	172.46	5	2.18E-35
0056<br>k02_allo_linwt1add_pre---_-------_FO--	BaseFO	.	7967.924	.	.	.

Difference in OFV (dOFV) = abs(Test model – Reference model)

Supplementary Table S6: Selection steps for MO compared with EPO-R elimination models

Model Number Model Identifier	Test Model	Reference Model	Alternate Hypothesis	Conclusion
0059 k02_allo_linwt1add _pre---_-------_----	BaseFOMO	.	.	.
0057 k02_allo_linwt1add _preall_EPO-R_CL_conc	0057	BaseFOMO	MO elimination better than EPO-R elimination from plasma using theory based allometric clearance	Accept
0058 k02_allo_linwt1add _pre---_EPO-R_kp	0058	BaseFOMO	MO elimination better than EPO-R elimination from plasma using empirical allometric elimination rate constant	Accept

Supplementary Table S7: Selection steps for rEPO pharmacokinetic and estimated weight model

Model Number and identifier	Test Model	Reference Model	Alternate Hypothesis	Conclusion
0092 k02_allo_linwt1add _aiHI_PCAlin_FOMO	Final	AICI	FOMO linear maturation better than no maturation	Accept
0095 k02_allo_linwt1add _aiHI_Tover_Vmax	.	Final	FOMO linear maturation better than rEPO induction effect of Vmax turnover	Accept
0088 k02_allo_linwt1add _aiCI_-------_----	AICI	BaseFOMO	Asphyxia, ischemia, hypothermia+ischemia effect on CL,V1,Q,V2,Vmax,km better than no effect	Accept
0059 k02_allo_linwt1add _pre---_-------_----	BaseFOMO	BaseFO	FO plus MO elimination better than FO elimination alone	Accept
0026 k02_allo_PostMwt--_pre---_------_----	.	BaseFOMO	Estimated weight better than post-mortem weight	Accept
0056 k02_allo_linwt1add _pre---_-------_FO--	BaseFO	.	.	.

Supplementary Table S8: Timeline of blood sample collection for rEPO plasma concentration measurements in the near-term ischemia-vehicle, sham-ischemia-rEPO, ischemia-rEPO, ischemia-hypothermia-rEPO, and preterm asphyxia-vehicle, asphyxia-rEPO 5000 IU infusion, asphyxia-rEPO 2000 IU infusion and asphyxia-rEPO bolus 5000 IU groups.

Study protocol	Fetal arterial blood sampling time for rEPO measurements
Near-term<br>Sham-ischemia rEPO<br>Ischemia-rEPO<br>Ischemia-hypothermia-rEPO<br>(20000 IU, 3333.2 IU/h infusion)	1 hour pre-EPO, 1, 3, 21, 45 and 69 hours during rEPO infusion, 24, 48, 72 and 96 hours after the end of infusion
Preterm<br>(5000 IU, 833.3 IU/h infusion)	1 hour pre-rEPO, 1.5, 3.5, 5.5, 23.5, 47.5 and 71.5 hours during rEPO infusion.
Preterm<br>(2000 IU, infusion at 520 IU/h)	1 hours pre-rEPO, 30 minutes, 6, 24, 48 and 66 hours during rEPO infusion, and 1, 6 and 24 hours after the end rEPO infusion
Preterm (5000 IU repeated bolus doses)	1 hour pre-rEPO, 30 minutes, 3, 6, 17, 24 hours after the second bolus injection of rEPO, and 1 hour pre-EPO and 30 minutes post-rEPO samples were collected for the first and third dose of rEPO.

Supplementary Material Pharmacokinetic Model

NM-TRAN Control Stream for rEPO pharmacokinetics with size, maturation and estimation of fetal weight

```
$PROB EPO IN PRE-TERM AND TERM SHEEP

$INPUT
ID ISPRE PROTOCOL_EVENT=DROP TIME_PCA PCA_DAY TIME AMT RATE DUR DVID DV MDV
MDV_BLQ LLOQ PM_PCA WTPM COOL ISCH ASPH BOLUS LONGINF OCC
;DVIDAMT=0, DVIDCONC=1, DVIDWT=2

$DATA ..\..\Data\epo_PK_PCAh.csv
IGNORE (LLOQ.GT.0)

$EST METHOD=COND INTER NSIG=3 SIGL=9
MAX=50000 NOABORT
PRINT=1 MSFO=EPI.MSF
$COV

$THETA
;Reference group is without ischemia|asphyxia or hypothermia (5 near-term plus 1 preterm)
(0.001,0.471,) ; POP_CL L/h/70kg
(0.001,4830.,) ; POP_Vmax IU/h/70kg
(0.001,441.,) ; POP_Km IU/L
(0.001,7.67,) ; POP_V L/70kg
(0.001,0.379,) ; POP_Q L/h/70kg
(0.001,13.4,) ; POP_V2 L/70kg
(0,0.211,) ; RUV_PROP
(0.001,5.08,) ; RUV_ADD IU

; asphyxia (placental artery constriction for 25 mins, preterm group only; this is equivalent to preterm
because only 1 preterm had sham asphyxia)
(0.001,1.03,4)  ; POP_FV1ASP
(0.001,0.419,4)  ; POP_FV2ASP
(0.001,0.57,4)  ; POP_FQASP
(0.01,1.39,4)  ; POP_FCLASP
(0.01,2.04,10)  ; POP_FVmASP
(0.01,2.89,20)  ; POP_FKmASP

; cerebral ischemia (carotid artery obstruction for 30 mins, near-term group only)
(0.001,0.872,4)  ; POP_FV1ISC
(0.001,1.2,4)  ; POP_FV2ISC
(0.001,1.79,4)  ; POP_FQISC
(0.01,1.51,4)  ; POP_FCLISC
(0.01,0.505,10)  ; POP_FVmISC
(0.01,0.85,20)  ; POP_FKmISC

; cerebral ischemia followed 3 h later by hypothermia for 69 h
(0.001,1.32,4)  ; POP_FV1COOL
```

```
(0.001,1.17,4)  ; POP_FV2COOL
(0.001,1.33,4)  ; POP_FQCOOL
(0.01,1.2,4)  ; POP_FCLCOOL
(0.01,0.775,10)  ; POP_FVmCOOL
(0.01,0.884,20)  ; POP_FKmCOOL

; time varying Vmax
(0,0,10000) FIX ; POP_EmaxVm fold
(0,1.,) FIX ; POP_C50Vm 1/(kIU/L)
(0.001,1.,10) FIX ; POP_HillVm
(0,1.,500)  FIX ; POP_MTT h

; maturation of elimination
0.0194 ; POP_FPCAFO PCA 1/day
0.0396 ; POP_FPCAMO PCA 1/day

; time varying WT
(0.25,1.5,) ; POP_WT0_PRE kg
(1,3.82,) ; POP_WT0_NEAR kg
(0,0.0716,) ; POP_WT_SLOPE ; kg/day
(0.001,0.367,) ; RUV_ADD_WT kg

$OMEGA BLOCK(3)
0.00054 ; PPV_CL
0.00973 0.27 ; PPV_Vmax
0.0249 0.488 1.16 ; PPV_Km
$OMEGA BLOCK(1)
0.0447 ; PPV_V
$OMEGA BLOCK(1)
0. FIX ; PPV_Q
$OMEGA BLOCK(1)
0. FIX ; PPV_V2
$OMEGA BLOCK(1)
0. FIX ; PPV_MTT

$OMEGA BLOCK(1)
0.00216 ; PPV_WT0
$OMEGA BLOCK(1)
0.528 ; PPV_WT_SLOPE

$SIGMA
1. FIX ; EPS1

$SUBR ADVAN13 TOL=9
$MODEL
COMP=(CENTRAL)
COMP=(PERIPHERAL)
COMP=(AUCT)
;COMP=(EFFECT)
;COMP=(TRANSIT1)
```

```
$PK
OBS=DV
IF (ASPH.EQ.1) THEN
  FV1ASP=THETA(9)
  FV2ASP=THETA(10)
  FQASP=THETA(11)
  FCLASP=THETA(12)
  FVMASP=THETA(13)
  FKMASP=THETA(14)
ELSE
  FV1ASP=1
  FV2ASP=1
  FQASP=1
  FCLASP=1
  FVMASP=1
  FKMASP=1
ENDIF

IF (ISCH.EQ.1.AND.COOL.NE.1) THEN ; Ischemia alone
  FV1ISC=THETA(15)
  FV2ISC=THETA(16)
  FQISC=THETA(17)
  FCLISC=THETA(18)
  FVMISC=THETA(19)
  FKMISC=THETA(20)
ELSE
  FV1ISC=1
  FV2ISC=1
  FQISC=1
  FCLISC=1
  FVMISC=1
  FKMISC=1
ENDIF

IF (ISCH.EQ.1.AND.COOL.EQ.1) THEN ; Ischemia and hypothermia
  FV1COOL=THETA(21)
  FV2COOL=THETA(22)
  FQCOOL=THETA(23)
  FCLCOOL=THETA(24)
  FVMCOOL=THETA(25)
  FKMCOOL=THETA(26)
ELSE
  FV1COOL=1
  FV2COOL=1
  FQCOOL=1
  FCLCOOL=1
  FVMCOOL=1
  FKMCOOL=1
ENDIF

; estimation of weight
```

```
IF (ISPRE.EQ.1) THEN
  GRP_WT0=THETA(33)
ELSE
  GRP_WT0=THETA(34)
ENDIF
GRP_WT_SLOPE=THETA(35)/24 ; kg/day -> kg/h
WT0=GRP_WT0*EXP(ETA(8))
WT_SLOPE=GRP_WT_SLOPE*EXP(ETA(9))
WTKG=WT0+WT_SLOPE*TIME

; maturation of elimination
GEST_DAY=TIME_PCA/24
SLOPE_FO=THETA(31)
SLOPE_MO=THETA(32)
FMATFO=(1+SLOPE_FO*(GEST_DAY-120))
FMATMO=(1+SLOPE_MO*(GEST_DAY-120))


GRP_V=THETA(4)*FV1ASP*FV1ISC*FV1COOL*(WTKG/70)
GRP_CL=THETA(1)*FCLASP*FCLISC*FCLCOOL*(WTKG/70)**0.75
GRP_V2=THETA(6)*FV2ASP*FV2ISC*FV2COOL*(WTKG/70)
GRP_Q=THETA(5)*FQASP*FQISC*FQCOOL*(WTKG/70)**0.75
GRP_VMAX=THETA(2)*FVMASP*FVMISC*FVMCOOL*(WTKG/70)**0.75
GRP_KM=THETA(3)*FKMASP*FKMISC*FKMCOOL


CL=GRP_CL*EXP(ETA(1))
VMAX=GRP_VMAX*EXP(ETA(2))
KM=GRP_KM*EXP(ETA(3))
V1=GRP_V*EXP(ETA(4))
Q=GRP_Q*EXP(ETA(5))
V2=GRP_V2*EXP(ETA(6))
;D1=DURH

EMAXVM=THETA(27)
C50VM=THETA(28)*1000 ; 1/(kIU/L) - > 1/(IU/L)
HILLVM=THETA(29)

GRP_MTT=THETA(30)
MTT=GRP_MTT;*EXP(PPV_MTT)
NTR=1 ; Number of transit compartments
KTR=(NTR+1)/MTT ; NTR plus effect cmt

S1=V1

$DES
DCE=A(4) ; IU/L
IF (DCE.GT.0) THEN
  DPD=0; EmaxVM/(1+(DCE/C50VM)**(-HILLVM))
ELSE
  DPD=0
```

```
ENDIF
DVMAXT=VMAX*(1+DPD)
C1=A(1)/V1
C2=A(2)/V2
DCLMO=DVMAXT/(KM+C1)
DADT(1)= Q*C2 - (FMATFO*CL+FMATMO*DCLMO)*C1
DADT(2)= Q*(C1 - C2)
DADT(3)=C1
;DADT(4)=KTR*(A(5)-DCE) ; effect cmt
;DADT(5)=KTR*(C1-A(5)) ; first transit cmt

$ERROR
CONC=A(1)/V1
IF (DVID.LE.1) THEN
  PROP=CONC*THETA(7)
  ADD=THETA(8)
  SD=SQRT(PROP*PROP + ADD*ADD)
  Y=CONC+SD*ERR(1)
ENDIF
IF (DVID.EQ.2) THEN
  Y=WTKG+THETA(36)*ERR(1)
ENDIF

CE=A(4)
IF (CE.GT.0) THEN
  PD=0 ; EmaxVM/(1+(CE/C50VM)**(-HILLVM))
ELSE
  PD=0
ENDIF
VMAXT=VMAX*(1+PD)
CLMO=VMAXT/(KM+CONC)
CLTOT=CL+CLMO
MRT=(V1+V2)/CLTOT
AUC=A(3) ; AUC 0-TIME IU/L*h

$TABLE ID TIME AMT RATE DUR ISPRE WTPM PCA_DAY
CL VMAX VMAXT PD CLMO CLTOT V1 Q V2 WT0 WT_SLOPE
MRT AUC CONC WTKG DVID Y
ONEHEADER NOPRINT FILE=pk.fit
```
